# Development of a laboratory test using stem cuttings to measure resistance to foot rot disease caused by *Diaporthe destruens* in sweetpotato

**DOI:** 10.1270/jsbbs.23072

**Published:** 2024-06-20

**Authors:** Hiroaki Tabuchi, Akira Kobayashi, Yukari Kawata, Yoshihiro Okada, Yuki Ohdaira Kobayashi

**Affiliations:** 1 Kyushu Okinawa Agricultural Research Center, NARO, Miyakonojo, Miyazaki 885-0091, Japan; 2 Kyushu Okinawa Agricultural Research Center, NARO, Itoman, Okinawa 901-0336, Japan

**Keywords:** foot rot, *Diaporthe destruens*, sweetpotato, laboratory test, stem, resistance

## Abstract

Over the last several years, foot rot caused by *Diaporthe destruens* has become the most destructive sweetpotato disease in the southernmost region of Japan. Breeding of cultivars resistant to foot rot is required for effective and low-cost management. Field tests are often used to evaluate resistance of cultivars, but this approach has several limitations, including a long test period of several months and the requirement of field isolation and labor-intensive procedures. To minimize these issues, we have developed an easier and faster laboratory method using stem cuttings for the resistance test by optimizing four parameters: the number of unfolded leaves per cuttings, the positions of stems from which a cutting was prepared, the adequate number of culture days after inoculation, and the density of conidia of *D. destruens* at inoculation. Significant correlation was detected between the resistance indices of the laboratory test and the field test, namely, the length of the rotted part of a stem and the proportion of the plants rotted at the basal part of a stem, respectively. These results indicated that the laboratory test could indirectly evaluate the foot rot resistance of sweetpotato stems in the field and will be helpful to breed resistant cultivars.

## Introduction

Foot rot, which is caused by *Diaporthe destruens* (Harter) Hirooka, Minosh. & Rossman, is a destructive fungal disease affecting sweetpotato [*Ipomoea batatas* (L.) Lam.]. It causes rotting of stems and storage roots, resulting in significant loss of commercial yields (Clark and Moyer 2013). Primary infection of *D. destruens* to sweetpotato plants is thought to occur in nurseries or fields. In nurseries, cuttings prepared from seed roots infected by *D. destruens* can be infected. In nurseries and fields, stem cuttings can be infected from plant debris in the soil because *D. destruens* can overwinter in plant debris (Clark and Moyer 2013, Kobayashi 2022). Secondary infection is thought to occur as follows: *D. destruens* grows in a stem of an infected plant and forms pycnidia on the surface of the stem. Conidia emerging from the pycnidia are washed away by the rain and spread in fields, and then the fungus infects stems of uninfected plants (Kobayashi 2022). After infection of the stems, *D. destruens* grows down into the storage roots. During the storage period of storage roots, uninfected storage roots can be infected through contact with infected storage roots.

Foot rot was first reported in United States (Harter 1913) but is no longer a common problem there (Clark and Moyer 2013). However, foot rot spread to Brazil in the 1990s (Lopes *et al.* 1994), while in Asia, foot rot was observed in Taiwan in 2008 (Lin *et al.* 2017), China in 2014 (Gai *et al.* 2016), and Korea in 2019 (Paul *et al.* 2019). In Japan, foot rot was first observed in the Okinawa and Kagoshima prefectures of Kyushu, the southern part of Japan, in 2018, and then spread to 27 prefectures by July 2022 (Kobayashi 2019, 2022, Maeda *et al.* 2022). In Kagoshima prefecture, the yield per 1000 m^2^ in 2021 decreased to 79% of the yield per 1000 m^2^ in 2017, and this was thought to be at least partly due to the influence of foot rot (Kagoshimaken satsumaimo denpun taisaku kyogikai 2022).

For effective and low-cost management of foot rot, breeding of resistant cultivars is required. The field test for foot rot resistance, in which cuttings prepared from the top part of stems of many sweetpotato cultivars are planted in a field naturally or artificially infested by *D. destruens*, is a common method for breeding resistant cultivars in routine breeding programs and is an excellent method to evaluate resistance as a close approximation of the actual resistance in farm fields. However, the method has some limitations: the density of the fungal pathogen is not even, the infested fields should be isolated to prevent contamination of the environment, the test is done only once a year, multiple years of testing are required because the evaluation changes depending on the weather, and the procedures are labor intensive. To minimize these issues, we here tried to develop a laboratory test for foot rot resistance using stem cuttings. To this end, we attempted to optimize the conditions of the laboratory test. First, we tested the number of unfolded leaves because the cuttings with fewer unfolded leaves withered more frequently for reasons other than foot rot. Next, to afford an adequate supply of cuttings, we investigated whether cuttings prepared from the middle part of stems could be used in the same way as the cuttings prepared from the top part of stems, which are commonly used for the field test. Then we researched the optimal number of incubation days after infection and the optimal density of conidia for inoculation. Finally, to examine whether the laboratory test could indirectly evaluate the resistance of stems of sweetpotato cultivars to foot rot under the field condition, we analyzed the correlation between the resistance index of the laboratory test, i.e., the length of the rotted part of a stem (LRS), and the resistance index of the field test, i.e., the proportion of the plants rotted at the basal part of a stem (PRS).

## Materials and Methods

### Fungal and plant materials

The foot rot pathogen used in this study was the F3 strain of *D. destruens*, which was originally isolated from fields in Miyazaki prefecture in Japan (Nomiyama *et al.* 2022). As sweetpotato cultivars, we used 22 cultivars whose resistance under field test conditions was previously evaluated by Kyushu Okinawa Agricultural Research Center, NARO (2023) (Table 1).

### Preparation of the conidial suspension

**Fig. 1.**
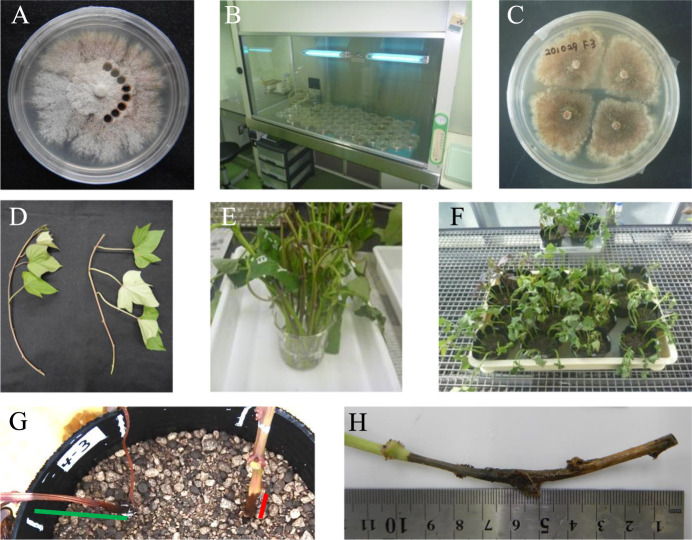
The method for the laboratory test using stem cuttings. A: Preparation of *D. destruens* blocks. Blocks of an SPDA plate with a diameter of 4 mm were cut out from the middle part of the spread area of the F3 strain after incubation at 25°C for 2 weeks. B, C: Pycnidium formation. Four blocks were put on a PDA plate and incubated under ultraviolet radiation at 25°C for 3 weeks for pycnidium formation. D: Preparation of stem cuttings. Cuttings were prepared from 15 to 25 cm of a top part (left) and a middle part (right) of sweetpotato stems. In this photo, there were 2 folded leaves and 3 unfolded leaves (left) and 3 unfolded leaves (right). All other leaves and petioles were removed. E: Inoculation. Cuttings were put in a beaker with conidial suspension for inoculation at room temperature for 1 h. F: Incubation. Cuttings were transferred to plastic pots with 7 cm depth soil and incubated at an average temperature 25°C in a green house. G: Simple evaluation. After incubation, we investigated the number of days until the withered part (green line) or the rotted part (red line) reached 8 cm or 2 cm from the soil surface, respectively. H: Detailed evaluation. After incubation, cuttings were washed, roots were removed, and the length of the rotted part of a stem was measured.

F3 was incubated on a sweetpotato dextrose agar (SPDA) plate [sweetpotato filtrate prepared by boiling 200 g of diced sweetpotato in 1000 ml of distilled water and filtering through gauze, 20 g of glucose (Fujifilm Wako, Tokyo) and 20 g of agar (Kanto Chemical, Tokyo); the pH was adjusted to 5.6] (Fujiwara *et al.* 2021) at 25°C for 2 weeks (Fig. 1). Four 4-mm-diameter blocks were then cut from the middle portion of the spread mycelium of F3 on the SPDA plate (Fig. 1A) and set on a potato dextrose agar (PDA) plate (Becton, Dickinson and Company, Franklin Lakes, NJ) in a plastic petri dish with a diameter of 9 cm. The PDA plate was incubated under ultraviolet radiation at 25°C for 3 weeks for pycnidium formation (Fig. 1B, 1C). After incubation, 20 ml of distilled water was added to one of the plates, and the plate was gently shaken to suspend the conidia. The conidial suspension was then transferred to another PDA plate to obtain a concentrated conidial suspension. The conidial suspension was finally collected in a glass beaker, and the density of conidia was adjusted with distilled water after counting the number of conidia using a hemocytometer. The conidial suspension was kept at room temperature and used for inoculation on the collection day, and was otherwise kept at 15°C and used on the next day.

### Basic method of the resistance test using stem cuttings

For the laboratory test, healthy cuttings of 15 to 25 cm long which contained at least 4 nodes were prepared from the top or middle part of sweetpotato stems grown at a nursery or a greenhouse (Fig. 1D).

For inoculation, approximately 3 cm of the bottom of the cuttings was dipped into the conidial suspension in a beaker at room temperature for 1 h (Fig. 1E). Distilled water was used instead of the conidial suspension for the negative control. Inoculated cuttings or uninoculated cuttings were transferred to a plastic pot with a diameter of 12 cm containing a 7 cm depth of sterilized ‘Kenbyo’ soil (Yaenogei, Saga, Japan) (Fig. 1F). The pots were incubated in a temperature-controlled greenhouse for up to 28 days under a temperature cycle of 23°C for 12 h and 27°C for 12 h, and were sufficiently watered every 2 to 3 days. The pots were shaded for the first 3 to 5 days to avoid wilting of the cuttings.

After incubation, for simple evaluation of the effect of two variables, i.e., the unfolded leaf number of a cutting and the position in the stem from which the cutting was prepared, on the resistance test, we investigated the number of days until the withered part of a stem due to a cause other than foot rot reached 8 cm from the soil surface until 21 days after inoculation (DAI) (Fig. 1G). There were no withered cuttings for which the withered part of the stem due to a cause other than foot rot had not reached 8 cm from the soil surface on 21 DAI. For other cuttings, we investigated the number of days until the rotted part of a stem due to foot rot reached 2 cm from the soil surface until 21 DAI (Fig. 1G). Because we distinguished between cuttings rotted in an above-ground stem and those withered in an above-ground stem by growing speeds and colors of symptoms of rotting and withering as described in the Discussion section, the cuttings on 21 DAI were sorted into three categories: cuttings for which the withered part of the stem due to a cause other than foot rot had reached 8 cm from the soil surface, cuttings for which the rotted part of the stem due to foot rot had reached 2 cm from the soil surface, and others. Others included cuttings for which the rotted part of the stem due to foot rot had not reached 2 cm from the soil surface, and cuttings with no symptoms of rotting or withering in an above-ground stem.

For detailed evaluation of the effect of two other variables, i.e., the number of incubation days after infection and the optimal density of conidia for inoculation, cuttings were washed, roots were removed, and LRS was measured (Fig. 1H).

### Examination of various conditions for the laboratory test

We selected ‘Koganesengan’ from the 22 cultivars described above to examine conditions for the resistance test against foot rot, because the resistance level of a stem of this cultivar was rather low in preliminary laboratory tests. In the tests to determine the number of unfolded leaves required to prevent a cutting from withering by a cause other than foot rot, the number of unfolded leaves in the cutting was adjusted by removing the lower leaves and petioles. Eight cuttings per 1 replication were used for each number of unfolded leaves. Because we could simultaneously conduct the tests to determine the number of unfolded leaves and the tests of the effect of the position in a stem from which a cutting was prepared, 4 of the 8 cuttings for each number of unfolded leaves were prepared from the top part of stems and 4 were prepared from the middle part of stems.

To determine the optimal DAI for the resistance test, we measured LRS using 4 cuttings of ‘Koganesengan’ on each of days 2, 5, 7, 9, 12, 14, 16, 19, 21, 23, 26 and 28 after inoculation with 10^6^ conidia/ml conidial suspension. We also measured LRS of 4 cuttings of ‘Tamaakane’, ‘Benihinata’, ‘Konaishin’, ‘Ayamurasaki’, ‘Daichinoyume’, ‘Konamizuki’, ‘Kokei No. 14’, ‘Benimasari’, ‘Beniharuka’, ‘Konahomare’, ‘Shiroyutaka’ and ‘Koganesengan’, on each of days 7, 14 and 21 after inoculation with 10^6^ conidia/ml conidial suspension.

To determine the optimal conidial concentration of *D. destruens* for the resistance test, we measured LRS on 21 DAI using 8 cuttings of each of ‘Tamaakane’, ‘Benihinata’, ‘Konaishin’, ‘Konamizuki’, ‘Benimasari’, ‘Shiroyutaka’, ‘Daichinoyume’ and ‘Koganesengan’ after an inoculation with 10^2^, 10^3^, 10^4^, 10^5^ and 10^6^ conidia/ml of conidial suspension.

### Final evaluation of the degree of resistance to foot rot in 22 cultivars

After examining various conditions for the laboratory test, we carried out tests to evaluate the degree of resistance to foot rot in 22 cultivars. Cuttings with 3 unfolded leaves prepared from the top or middle part of stems were inoculated with 10^4^ conidia/ml conidial suspension of *D. destruens*, and the LRS was measured on 21 DAI. For 1 cultivar, 3 to 4 cuttings were usually inoculated and 1 cutting was used as a negative control. Because there were rarely uninfected cuttings or withered cuttings due to causes other than foot rot among these inoculated cuttings, 1 replication of a test for 1 cultivar consisted of 2 to 4 infected cuttings inoculated at the same test. We carried out at least three replications of the test for each cultivar, and if the 95% confidence interval of the mean of LRS, determined as 1.96 × standard error (*SE*), was over 1.5 cm, we carried out additional replications. The final LRS of each cultivar was determined as the average of all replications. The statistical analysis was carried out by JMP (SAS Institute, Cary, NC).

### Correlation analysis between laboratory tests and field tests

We calculated the correlation between LRS of the laboratory tests and PRS in the field tests using PRS data from the Kyushu Okinawa Agricultural Research Center, NARO (2023).

## Results

### Effect of unfolded leaf number and position in stem cuttings on the resistance test

In preliminary laboratory tests using cuttings with fewer unfolded leaves, not only inoculated cuttings but also uninoculated control cuttings sometimes exhibited withering with the same symptoms. Because the symptom profile seemed to be different from that of foot rot, we considered that the withering of these cuttings was attributable to some cause other than foot rot (Fig. 1G, Supplemental Fig. 1), and we tested the number of unfolded leaves required to avoid withering of these stems by the presumably alternative cause. As shown in the 3^rd^ to 5^th^ lines from the top of each replication in Table 2, although the number of days until the withered part of a stem reached 8 cm from the soil surface was not affected by the number of unfolded leaves of cuttings in both replications, whether cuttings were withered or unwithered due to a cause other than foot rot was affected by the number of unfolded leaves of cuttings. More than half of the cuttings without unfolded leaves withered due to a cause other than foot rot, while about half of the cuttings with 1 and 2 unfolded leaves withered, and less than a quarter of the cuttings with 3 to 5 unfolded leaves withered when the cuttings were inoculated with 10^6^ conidia/ml conidial suspension.

Along with testing the number of unfolded leaves, to afford an adequate supply of cuttings, we also investigated whether the cuttings of the middle part of stems could be used in the same way as the cuttings of the top part of stems. In this examination, we focused only on the cuttings that were not withered due to causes other than foot rot (3 lines from the bottom of each replication in Table 2). Although the number of days until the rotted part of a stem reached 2 cm from the soil surface was significantly affected by the number of unfolded leaves of cuttings in replication 2, the number of days until the rotted part of a stem reached 2 cm from the soil surface was not significantly different between the cuttings of the top part of stems and those of the middle part of stems in both replications in Table 2. Also, in replication 1, the proportion of cuttings rotted due to foot rot was not significantly different between the cuttings prepared from the top part of stems and those prepared from the middle part.

From these results, we decided to use the cuttings with 3 unfolded leaves prepared from the top or middle part of stems for the resistance test, because the proportion of cuttings with withering due to causes other than foot rot was almost the same among the cuttings with 3, 4 or 5 unfolded leaves, fewer unfolded leaves could make for easier handling, and the number of days until the rotted part of a stem reached 2 cm from the soil surface was not significantly different between the top and middle parts of stems from which the cuttings were prepared.

### Effect of the incubation period after inoculation on the resistance test

We carried out two tests to determine the optimal DAI for the resistance test. First, we measured the LRS of ‘Koganesengan’ over 2 to 28 DAI. As shown in Supplemental Fig. 2, the relationship between DAI and LRS fit a linear model (*R*^2^ ≥ 0.88). This result indicated that LRS became larger as DAI increased. Next, we measured the LRS of 12 cultivars each on 7, 14 and 21 DAI. Supplemental Fig. 3 shows that the LRS in each cultivar linearly increased during the incubation (*R*^2^ ≥ 0.49). Among these 12 cultivars, ‘Tamaakane’ and ‘Koganesengan’ were considered the most resistant and susceptible cultivars because the mean LRS of ‘Tamaakane’ and ‘Koganesengan’ on 21 DAI was shortest and longest in 12 cultivars, respectively. The difference of mean LRS between these two cultivars became larger as DAI increased: 0.0, 5.1 and 9.4 cm on 7, 14 and 21 DAI, respectively. Although a larger difference of LRS between the resistant and susceptible cultivars would be advantageous for achieving an accurate estimation of the resistance of cultivars, we considered that the longer incubation required to achieve this difference would require more greenhouse space for the incubation, so we choose 21 DAI for the resistance test.

### Effect of the conidial concentration of *D. destruens* on the resistance test

Finally, we tested the optimal conidial concentration of *D. destruens* for the resistance test. We measured the LRS of cuttings of 8 cultivars inoculated with 10^2^ to 10^6^ conidia/ml of conidial suspension. The results in Table 3 demonstrate that the difference between maximum and minimal LRS become larger at the lower conidial concentration except for 10^2^ conidia/ml. The larger difference in LRS made it easier to distinguish between the resistant and susceptible cultivars. But the resistance tests become unstable when cuttings were inoculated at concentrations of 10^2^ and 10^3^ conidia/ml because the proportion of cuttings without foot rot symptom was more than 25.0%. Furthermore, higher concentrations of conidial suspension would require that more conidia be prepared. Thus, we decided to inoculate cuttings with a conidial suspension at 10^4^ conidia/ml concentration for the resistance test.

Based on the above, the laboratory resistance test was carried out as follows. Cuttings with 3 unfolded leaves prepared from the top or middle part of stems were inoculated with 10^4^ conidia/ml conidial suspension of *D. destruens*. LRS was measured on 21 DAI.

### Correlation between laboratory tests and field tests

Kyushu Okinawa Agricultural Research Center, NARO (2023) reported the PRS values of 15, 20 and 12 cultivars in field tests in 2020, 2021 and 2022, respectively (Table 4). In the present study, we conducted laboratory tests of the resistance of these sweetpotato cultivars to analyze the correlation between the LRS values yielded by our laboratory tests and the PRS values from the field tests of the Kyushu Okinawa Agricultural Research Center (Table 4, Supplemental Fig. 4). The LRS ranged from 3.7 to 7.0. On August 24^th^ in 2020 and on all investigated days in 2021 and 2022, significant correlations were observed between the LRS and PRS.

## Discussion

### Examination of the conditions for the resistance test

Because more unfolded leaves meant more troublesome handling, in our preliminary tests of resistance, we removed all unfolded leaves and petioles from the cuttings. However, the cuttings with fewer unfolded leaves withered more frequently due to causes other than foot rot (Table 2). One of the possible reasons for this withering would be a deficiency of available water due to evaporation from the cut surface of petioles. That is, the cuttings with fewer unfolded leaves and many cut surfaces would not have been able to simply close their stomata to prevent water evaporation. This hypothesis is supported by the fact that the withered portions of the cuttings were usually dry (Supplemental Fig. 1). Furthermore, although the position in stems from which the cuttings were prepared did not significantly affect the number of days until the withered part of a stem reached 8 cm from the soil surface, it significantly affected whether the cuttings were withered or unwithered due to causes other than foot rot (Table 2). It is unknown why the cuttings prepared from the top part of stems withered more frequently than those from the middle part of stems (Table 2). In any case, because withering due to causes other than foot rot occurred more quickly than rotting due to foot rot, it was not possible to accurately evaluate the effects of foot rot in the former case. Thus, we investigated how many unfolded leaves were needed in order to avoid withering due to causes other than foot rot. In these tests, we counted the number of the cuttings of which the withered part of a stem due to causes other than foot rot had reached 8 cm from the soil surface as well as the number of the cuttings for which the rotted part of a stem due to foot rot had reached more than 2 cm from the soil surface on 21 DAI. We were unable to observe the rotted part of stems less than 2 cm from the soil surface because the base part of the plants was shadowed and difficult to see. But we did observe the rotted stem portions beginning at more than 2 cm from the soil surface. According to these observations, the foot rot-affected portion of ‘Koganesengan’ stems grew at about 0.25 to 0.75 cm a day, and it rarely reached 8 cm from the soil surface until 21 DAI (data not shown). On the other hand, the portions of ‘Koganesengan’ stems withered by causes other than foot rot grew at a rate of about 4 cm a day, and these stems reached 8 cm from the soil surface by 21 DAI (data not shown). Thus, because we observed rotting and withering in above-ground stems almost every day, the 2 cm rotted part of stems due to foot rot and the 8 cm withered part of stems due to causes other than foot rot were easily distinguished.

Regarding the position in stems from which the cuttings were prepared, we further analyzed the difference between 77 and 37 cuttings prepared from the top part and the middle part of stems, respectively, in the 31 replications of ‘Koganesengan’ described in Table 4. There were no significant differences in the proportion of infected cuttings by foot rot, withered cuttings due to causes other than foot rot and cuttings with no symptoms of disease between the two stem regions, and there were also no significant differences in LRS between the 72 cuttings prepared from the top part of stems and 33 cuttings prepared from the middle portion (Table 5). This result supported that a cutting prepared from the middle part of stems can be used in the same manner as a cutting prepared from the top part of stems.

In the tests to determine an optimal DAI, the rotted portion of stems was rarely apparent until 5 DAI (Supplemental Fig. 2). Germination of conidia and subsequent increase in adequate mycelia, which might be necessary for stem rot, seemed to require a certain amount of time.

In the tests to determine the optimal conidial concentration, the mean LRS of 8 cultivars increased as the conidial concentration increased, with the exception of a conidial concentration of 10^2^ conidia/ml (Table 3). The LRS of each cultivar also became larger as the conidial concentration increased, with the exception of the conidial concentrations of 10^2^ conidia/ml and 10^3^ conidia/ml. Furthermore, uninfected cuttings were detected at the proportions of 25.0% and 29.7% after inoculation at the conidial concentrations of 10^2^ conidia/ml and 10^3^ conidia/ml, respectively. Infection seemed to be unstable at the conidial concentrations of 10^2^ conidia/ml and 10^3^ conidia/ml. These results indicated that the threshold of the conidial concentration at which almost all cuttings become infected was between 10^3^ conidia/ml and 10^4^ conidia/ml under our inoculating conditions. It should be noted that the proportion of the uninfected cuttings at low conidial concentration cannot be used as a resistance index of a stem in the laboratory test, because significant correlation was not detected between the proportion of the uninfected cuttings and LRS (10^2^ conidia/ml, *r* = –0.33, *p* = 0.42. 10^3^ conidia/ml, *r* = –0.54, *p* = 0.17).

### Correlation between the laboratory tests and field tests

The Kyushu Okinawa Agricultural Research Center, NARO (2023) evaluated the resistance to foot rot of 22 cultivars in field tests using 5 degrees of resistance to susceptible cultivars (R, Rʹ, I, Sʹ, S) (Tables 1, 4), based on the degree of severity of foot rot in stems and storage roots. The mean LRS of 8 resistant cultivars (R and Rʹ) was 5.0, and the mean LRS of 8 susceptible cultivars (S and Sʹ) was 6.5 (Table 4). Based on these results, we classified LRS into three groups, i.e., a resistant, an intermediate and a susceptible group, in which LRS of cultivars were under 5.0, 5.0 to 6.5, and over 6.5, respectively. To avoid confusion between the resistant and susceptible groups, we considered that the 95% confidence interval of the mean, 1.96 times the *SE*, should be less than 1.5 (i.e., 6.5 minus 5.0), meaning that the *SE* should be less than 0.77. Thus, we carried out at least three replications of the resistance test of a stem for each cultivar, and then, if the product of 1.96 and the *SE* was greater than 1.5, we carried out additional replications. The number of replications of ‘Koganesengan’ and ‘Tamaakane’ increased because we used them as the control cultivars for susceptibility and resistance, respectively, in almost all tests (Table 4). We sometimes used ‘Benihinata’ and ‘Tamayutaka’ as the secondary control cultivars for resistance, resulting in additional replications.

The correlation coefficients between LRS in the laboratory tests and PRS in the field tests were higher on August 24^th^ in 2020 and August 20^th^ in 2021 (Table 4, Supplemental Fig. 4). However, the correlation coefficients were lower on October 6^th^ in 2020 and October 12^th^ in 2021. This was probably because many of the cultivars were ‘saturated’ with an infection proportion of 100% at the stem base in October. And the lower correlation coefficients in the early July of 2020 and 2021 would have been caused by the small difference in the PRS among the cultivars. Because the spread of foot rot disease in the fields was slower in 2022 than in 2020 and 2021 seemly due to influence from environmental factors, the correlation coefficient was lower in August than in October in 2022.

In this study we developed a laboratory test for resistance to foot rot using stem cuttings. This laboratory test can be used to investigate the resistance of the stems of sweetpotato cultivars throughout the year with no major influence from environmental factors. Furthermore, we could use LRS in the laboratory test as the index to evaluate PRS in the field test indirectly because significant correlations were observed between the LRS and PRS on August 24^th^ in 2020 and on all investigated days in 2021 and 2022 (Table 4). However, this laboratory test has limitations. One of the drawbacks is the narrow range of LRS, which makes it difficult to categorize LRS into five groups for the resistance evaluation in the field test.

In the breeding program, the laboratory test established in this study has been attempted to use for preliminary selection of resistant cultivars to foot rot disease. In winter, we evaluated the resistance of shoots sprouted from storage roots which were harvested in autumn. In spring, plants which were evaluated as resistant using the laboratory test were planted in the field naturally or artificially infested by *D. destruens* for the field resistant test. The laboratory test seems to be useful for effective breeding of resistant cultivars to foot rot. We have already been using the laboratory test to evaluate the resistance of F_1_ plants for genetic analysis to find QTLs for resistance to foot rot, too. We are also searching for genetic resources highly resistant to foot rot using this laboratory test. However, we have not yet found such a cultivar for which the LRS is almost zero. So, we can only evaluate the ‘relative’ resistance to foot rot.

## Author Contribution Statement

HT conducted the laboratory tests, analyzed the data and drafted the manuscript. YOK contributed to the preparation of *D. destruens*. YK and YO contributed to the design of the preliminary tests of resistance. HT and AK prepared the sweetpotato cultivars. All authors contributed to the article and approved the submitted version.

## Supplementary Material

Supplemental Figures

## Figures and Tables

**Table 1.** Cultivars used in this study

Cultivars	Usage	Resistance evalutation*^a^*
Amahazuki	table use	I
Beniazuma	table use	Sʹ
Beniharuka	table use	S or Sʹ
Benihinata*^b^*	table use	R
Benimasari	table use	Rʹ
Beniotome	table use	I
Kokei No. 14	table use	Sʹ
Suzuhokkuri	table use	Rʹ
Daichinoyume	raw materials	S
Koganesengan	raw materials	Sʹ
Konahomare	raw materials	S
Konaishin	raw materials	Rʹ
Konamizuki	raw materials	S
Michishizuku	raw materials	Rʹ
Satsumamasari	raw materials	I
Shiroyutaka	raw materials	I or Sʹ
Ayamurasaki	processed foods	I
Benihayato	processed foods	R
Murasakimasari	processed foods	I
Okikogane	processed foods	R
Tamaakane	processed foods	R
Tamayutaka	processed foods	I

*^a^* The resistance evaluation to foot rot was based on the Kyushu Okinawa Agricultural Research Center, NARO (2023). The resistance evaluation categories from high resistance to low resistance in field tests were as follows: R: resistant; Rʹ: less resistant; I: intermediate; Sʹ: less susceptible; S: susceptible. *^b^* ‘Benihinata’ is described as ‘Kyushu No. 201’ at the Kyushu Okinawa Agricultural Research Center, NARO (2023).

**Table 2.** The effects of the number of unfolded leaves and the position in a stem from which the cutting was prepared on withering and rotting of cuttings

Replication 1	Number of unfolded leaves								Position in stems		
Cuttings	0	1	2	3	4	5			Top	Middle	
Number of withered cuttings due to other than foot rot*^a^*	7	4	4	2	1	2	*p* < 0.01*^g^*		16	4	*p* < 0.01*^g^*
Number of unwithered cuttings due to other than foot rot*^b^*	1	4	4	6	7	6			8	20	
Mean day when the withered part of stem reached 8 cm*^c^*	12.7	12.0	8.5	9.5	14.0	11.5	*p* = 0.43*^h^*		11.1	12.5	*p* = 0.41*^h^*
Number of rotted cuttings due to foot rot*^d^*	1	3	0	6	7	4	*p* = 0.38*^i^*		8	13	*p* = 0.07*^l^*
Number of unrotted cuttings due to foot rot*^e^*	0	1	4	0	0	2			0	7	
Mean day when the rotted part of stem reached 2 cm*^f^*	16.0	15.0	—	17.3	18.0	15.5	*p* = 0.89*^j^*		15.9	16.2	*p* = 0.73*^j^*
											
Replication 2	Number of unfolded leaves								Position in stems		
Cuttings	0	1	2	3	4	5			Top	Middle	
Number of withered cuttings due to other than foot rot*^a^*	5	4	3	1	0	0	*p* < 0.01*^g^*		10	3	*p* = 0.01*^g^*
Number of unwithered cuttings due to other than foot rot*^b^*	3	4	5	7	8	8			14	21	
Mean day when the withered part of stem reached 8 cm*^c^*	11.6	10.8	12.0	14.0	—	—	*p* = 0.11*^h^*		11.5	12.0	*p* = 0.45*^h^*
Number of rotted cuttings due to foot rot*^d^*	3	4	5	7	8	8	n.a.*^k^*		14	21	n.a.*^k^*
Number of unrotted cuttings due to foot rot*^e^*	0	0	0	0	0	0			0	0	
Mean day when the rotted part of stem reached 2 cm*^f^*	12.3	11.0	11.6	11.6	13.9	14.0	*p* = 0.03*^j^*		11.9	13.1	*p* = 0.10*^j^*

Eight cuttings of ‘Koganesengan’ for each number of unfolded leaves were inoculated with 10^6^ conidia/ml conidial suspension. Of the 8 cuttings, 4 were prepared from the top part of stems and 4 were prepared from the middle part of stems. *^a^* The number of cuttings of which the withered part of a stem due to causes other than foot rot had reached 8 cm from the soil surface on 21 days after inoculation. *^b^* The total of the number of the cuttings of which the rotted part of a stem due to foot rot had reached 2 cm from the soil surface, the number of the cuttings of which the rotted part of a stem due to foot rot had not reached 2 cm from the soil surface, and the number of the cuttings with no symptoms of rotting and withering in the stem above the soil surface on 21 days after inoculation. There were no withered cuttings of which the withered part of a stem due to causes other than foot rot had not reached 8 cm from the soil surface on 21 days after inoculation. *^c^* Mean day when the withered part of a stem of the withered cuttings reached 8 cm from the soil surface. Data of unwithered cuttings were not used for calculating the mean day. *^d^* The number of the cuttings of which the rotted part of a stem due to foot rot had reached 2 cm from the soil surface on 21 days after inoculation. *^e^* The total of the number of the cuttings of which the rotted part of a stem due to foot rot had not reached 2 cm from the soil surface, and the number of the cuttings with no symptom of rotting and withering in a stem above the soil surface on 21 days after inoculation. *^f^* Mean day when the rotted part of a stem of the rotted cuttings reached 2 cm from the soil surface. Data of unrotted cuttings were not used for calculating the mean day. *^g^* Logistic analysis to determine whether cuttings were withered or unwithered using the number of unfolded leaves and the position in stems. *^h^* Multiple regression analysis of the number of days when the withered part of a stem reached 8 cm using the number of unfolded leaves and the position in stems. *^i^* Logistic analysis to determine whether cuttings were rotted or unrotted using the number of unfolded leaves. Logistic analysis to determine whether cuttings were rotted or unrotted using the number of unfolded leaves and the position in stems was not done because of the result of the goodness-of-fit test (*p* = 0.02). *^j^* Multiple regression analysis of the number of days when the rot reached 2 cm using the number of unfolded leaves and the position in stems. *^k^* Statistical analysis could not be done because the number of unrotted cuttings was 0. *^l^* Fisher’s exact test. Logistic analysis to determine whether cuttings were rotted or unrotted using the number of unfolded leaves and the position in stems was not done because of the result of the goodness-of-fit test (*p* = 0.02).

**Table 3.** Length of the rotted part of a stem (LRS) inoculated with various conidial concentrations

Cultivar	Resistance evalutation*^w^*	Conidial concentration (conidia/ml)														Mean LRS
		10^2^			10^3^			10^4^			10^5^			10^6^		
		LRS ± *SE*	uninfected cuttings		LRS ± *SE*	uninfected cuttings		LRS ± *SE*	uninfected cuttings		LRS ± *SE*	uninfected cuttings		LRS ± *SE*	uninfected cuttings	
Benihinata*^xy^*	R	5.1 ± 0.3 ab	3		3.9 ± 0.9 a	5		4.2 ± 0.1 a	1		6.3 ± 0.0 ab	0		7.4 ± 0.5 b	0	5.4
Tamaakane*^x^*	R	2.9 ± 0.1 a	3		2.5 ± 1.5 a	6		4.4 ± 1.1 a	0		5.8 ± 0.3 a	0		7.8 ± 1.0 a	0	4.7
Konaishin*^x^*	Rʹ	5.6 ± 0.3 b	0		3.3 ± 0.2 a	0		5.7 ± 0.3 b	0		6.3 ± 0.3 b	0		8.1 ± 0.2 c	0	5.8
Benimasari*^x^*	Rʹ	6.4 ± 1.1 a	0		6.1 ± 0.1 a	1		5.9 ± 0.0 a	0		7.2 ± 0.2 a	0		9.2 ± 0.7 a	0	6.9
Shiroyutaka*^x^*	I or Sʹ	4.7 ± 0.2 a	2		4.9 ± 0.4 a	3		6.6 ± 0.1 ab	0		8.5 ± 0.1 b	0		7.8 ± 0.7 b	0	6.5
Koganesengan*^x^*	Sʹ	6.4 ± 1.4 ab	2		5.8 ± 0.3 a	1		7.3 ± 0.0 ab	1		7.4 ± 0.2 ab	0		9.6 ± 0.3 b	0	7.3
Daichinoyume*^x^*	S	6.1 ± 0.1 a	4		5.8 ± 1.5 a	1		6.4 ± 0.3 a	0		7.4 ± 0.3 a	0		9.1 ± 0.2 a	0	7.0
Konamizuki*^x^*	S	5.5 ± 0.3 ab	2		3.3 ± 1.2 a	2		7.3 ± 0.2 bc	0		8.2 ± 0.7 bc	0		10.2 ± 0.3 c	0	6.9
Mean LRS*^x^*		5.3 ± 0.4 ab			4.4 ± 0.5 a			6.0 ± 0.4 bc			7.1 ± 0.3 c			8.6 ± 0.3 d		6.3
Max. LRS - min. LRS		3.5			3.6			3.1			2.8			2.8		3.2
Total number of uninfected cuttings*^z^*			16**			19**			2**			0**			0**	
Propotion of uninfected cuttings (%)			25.0			29.7			3.1			0.0			0.0	

Four cuttings of each cultivar per 1 replication were inoculated with each indicated concentration of conidial suspension. The mean LRS of 2 replications was calculated. *^w^* The resistance evaluation categories were described in Table 1. *^x^* LRS of a single cultivar or mean LRS of 8 cultivars was compared among 5 conidial concentrations by Tukey-Kramer test. Results with the same letter (a, b, or c) were not significantly different (*p* < 0.05). *^y^* ‘Benihinata’ is described as ‘Kyushu No. 201’ at the Kyushu Okinawa Agricultural Research Center, NARO (2023). *^z^* The number of uninfected cuttings was compared among 5 conidial concentrations by χ^2^ test. **p < 0.01.

**Table 4.** Correlation between the proportion of the plants rotted at the basal part of a stem (PRS) in field tests and the length of the rotted part of a stem (LRS) in laboratory tests

Cultivars	Laboratory test		Field test																				
	LRS ± *SE* (replication)		PRS in 2020 (%)								PRS in 2021 (%)								PRS in 2022 (%)				Resistance evalutation*^a^*
			Jul. 13	Jul. 29	Aug. 13	Aug. 24	Sep. 11	Sep. 25	Oct. 6		Jul. 5	Jul. 19	Aug. 3	Aug. 20	Sep. 2	Sep. 19	Oct. 12		Aug. 23	Sep. 20	Oct. 7	Oct. 17	
Benihayato	3.7 ± 0.1 (4)										0.0	1.1	2.3	2.3	1.1	5.7	4.6						R
Okikogane	4.2 ± 0.2 (4)										0.0	0.0	2.2	4.4	8.9	10.0	17.8						R
Ayamurasaki	4.7 ± 0.4 (7)		1.1	4.4	13.3	17.8	25.6	58.9	90.0		3.6	11.7	18.5	31.0	46.5	73.1	95.4		5.6	20.8	33.3	51.4	I
Benihinata*^b^*	4.8 ± 0.3 (15)																		0.0	0.0	2.8	5.6	R
Murasakimasari	4.9 ± 0.3 (4)		0.0	4.5	9.0	11.2	31.5	66.3	84.3		0.0	1.7	10.4	37.9	56.8	73.7	96.4						I
Tamaakane	5.0 ± 0.2 (35)		1.1	3.3	2.2	1.1	3.3	5.6	8.9		1.2	2.3	2.3	3.5	4.7	9.4	10.6		1.4	2.8	2.8	4.2	R
Michishizuku	5.3 ± 0.4 (5)										0.0	1.7	1.7	11.7	23.3	36.7	76.7		5.6	16.7	29.2	38.9	Rʹ
Suzuhokkuri	5.4 ± 0.2 (4)										0.0	4.5	19.0	20.1	23.6	30.2	43.8						Rʹ
Tamayutaka	5.4 ± 0.3 (13)		5.6	5.6	7.8	11.1	14.4	35.6	43.3		2.2	3.3	8.9	30.3	39.3	56.2	71.0						I
Kokei No. 14	5.4 ± 0.4 (9)		8.9	18.9	34.4	42.2	58.9	77.8	92.2		10.0	24.6	34.8	59.5	75.4	95.6	100.0		16.7	38.9	56.9	70.8	Sʹ
Satsumamasari	5.6 ± 0.4 (8)		3.3	5.6	13.3	21.1	36.7	57.8	70.0		2.2	8.9	23.3	45.6	60.0	77.8	96.7						I
Konaishin	5.6 ± 0.4 (8)		0.0	1.1	2.2	8.9	13.3	20.0	23.3		2.2	6.7	8.9	16.9	28.1	41.8	50.5		4.2	8.3	9.7	11.1	Rʹ
Amahazuki	5.9 ± 0.3 (4)																		0.0	22.2	41.7	52.8	I
Beniharuka	6.1 ± 0.4 (4)		7.8	21.1	25.6	38.9	57.8	82.2	97.8		6.7	14.4	55.6	82.2	88.9	97.8	100.0		36.1	72.2	84.7	91.7	S or Sʹ
Beniotome	6.2 ± 0.4 (4)										3.4	8.5	20.3	45.7	55.9	79.6	91.5						I
Benimasari	6.3 ± 0.2 (9)		1.1	3.4	2.2	4.5	9.0	16.9	56.2		3.3	10.1	19.0	26.9	31.4	47.3	71.8		4.2	14.1	25.5	31.8	Rʹ
Beniazuma	6.4 ± 0.4 (5)		6.7	25.6	32.2	34.4	47.8	83.3	97.8		4.4	20.0	33.3	61.1	75.6	93.3	100.0						Sʹ
Konamizuki	6.6 ± 0.4 (6)		12.2	26.7	50.0	66.7	80.0	93.3	100.0		4.4	21.1	46.7	81.0	92.2	95.6	100.0						S
Shiroyutaka	6.6 ± 0.3 (4)		1.1	5.6	13.3	27.8	41.1	58.9	76.7		0.0	6.7	10.0	23.5	32.4	44.8	68.3		15.3	47.2	63.9	79.2	I or Sʹ
Daichinoyume	6.8 ± 0.4 (7)		20.0	53.3	77.8	91.1	96.7	100.0	100.0		14.4	52.2	81.1	93.3	100.0	100.0	100.0		50.0	86.1	100.0	100.0	S
Koganesengan	7.0 ± 0.2 (31)		1.1	8.9	17.8	25.6	38.9	65.6	86.7		7.8	16.7	45.6	70.0	90.0	96.7	100.0		16.7	59.7	83.3	88.9	Sʹ
Konahomare	7.0 ± 0.2 (9)		1.1	8.9	17.8	25.6	38.9	65.6	86.7		11.7	40.0	80.0	95.0	100.0	100.0	100.0						S
Mean	5.7		5.0	13.4	21.5	28.7	39.6	58.7	73.4		3.5	11.4	23.4	39.3	49.2	61.3	73.4		13.0	32.4	44.5	52.2	
Correlation coefficient*^c^*			0.33	0.46	0.46	0.52*	0.51	0.42	0.42		0.61**	0.65**	0.71**	0.75**	0.74**	0.70**	0.66**		0.59*	0.74**	0.76**	0.70*	

RPS was based on the Kyushu Okinawa Agricultural Research Center, NARO (2023). *^a^* The resistance evaluation categories were described in Table 1. Resistance evaluation showed a statistically significant correlation with LRS and all PRS calculated by replacing R, Rʹ, I or Sʹ, Sʹ, S or Sʹ and S with 1, 2, 3, 3.5, 4, 4.5 and 5, respectively. *^b^* ‘Benihinata’ is described as ‘Kyushu No. 201’ at the Kyushu Okinawa Agricultural Research Center, NARO (2023). *^c^* Correlation coefficients were calculated between RPS in field tests and LRS in laboratory tests. *p < 0.05. **p < 0.01.

**Table 5.** Statistical analyses of cuttings prepared from the top and middle part of stems of ‘Koganesengan’

	Position in stems		
	Top	Middle	
Number of infected cuttings by foot rot	72	33	
Number of withered cuttings due to other than foot rot	2	4	*p* = 0.10*^a^*
Number of cuttings with no symptoms of diseases	3	0	
LRS ± *SE*	7.0 ± 0.2	6.7 ± 0.2	*p* = 0.26*^b^*

Statistical analyses were done using the 77 and 37 cuttings prepared from the top and middle portions of stems, respectively, in the 31 replications of ‘Koganesengan’ described in Table 4. *^a^* χ^2^ test. *^b^* *t* test using 72 and 33 cuttings prepared from the top and middle part of stems, respectively.
