# Supplementary material for: Development of a laboratory test using stem cuttings to measure resistance to foot rot disease caused by Diaporthe destruens in sweetpotato
Source: Breed Sci. 2024 Jun 20;74(3):214–22. doi: 10.1270/jsbbs.23072 (PMC11561415; doi:10.1270/jsbbs.23072)
Supplement: Supplementary file 1 — Supplemental Figures [file 74_214-s1.pdf]

doi: 10.1270/jsbbs.23072

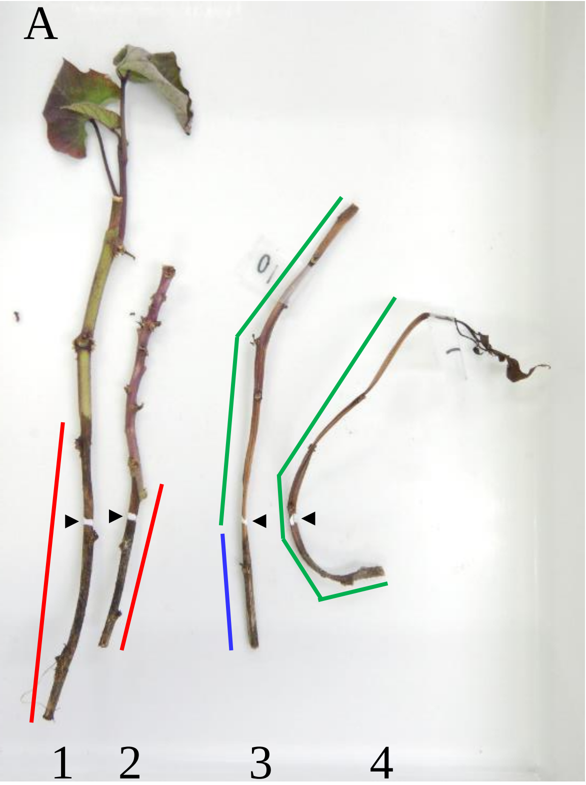

Supplemental Fig. 1 Cuttings rotted due to foot rot and withered for other reasons. A white line on a cutting and an arrowhead are located at the soil surface. A red line indicates the rotted part of a stem due to foot rot. A green line indicates the withered part of a stem due to causes other than foot rot. We could not distinguish between the rotted part and the withered part of stems indicated by the blue line. **1, 2**: Inoculated cuttings of ‘Koganesengan’ partially rotted only due to foot rot. The colors of the rotted part of a stem due to foot rot were generally black, dark brown and/or brown in the laboratory tests and the field tests. **3, 4**: An inoculated cutting (3) and an uninoculated control cutting (4) of ‘Koganesengan’ withered due to causes other than foot rot. The color of the withered part of a stem due to causes other than foot rot was generally light brown.

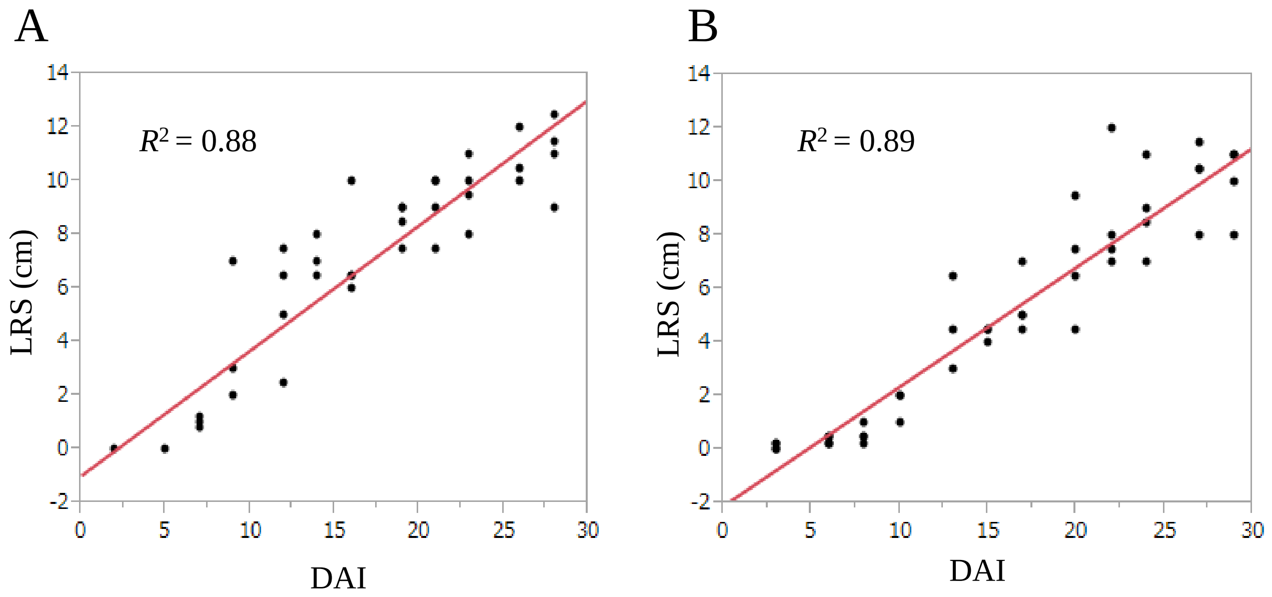

Supplemental Fig. 2 The relationship between DAI and LRS. LRS values were measured using 4 cuttings of ‘Koganesengan’ on each of days 2, 5, 7, 9, 12, 14, 16, 19, 21, 23, 26 and 28 after inoculation with 10^6 conidia/ml conidial suspension. **A**: Replication 1. **B**: Replication 2.

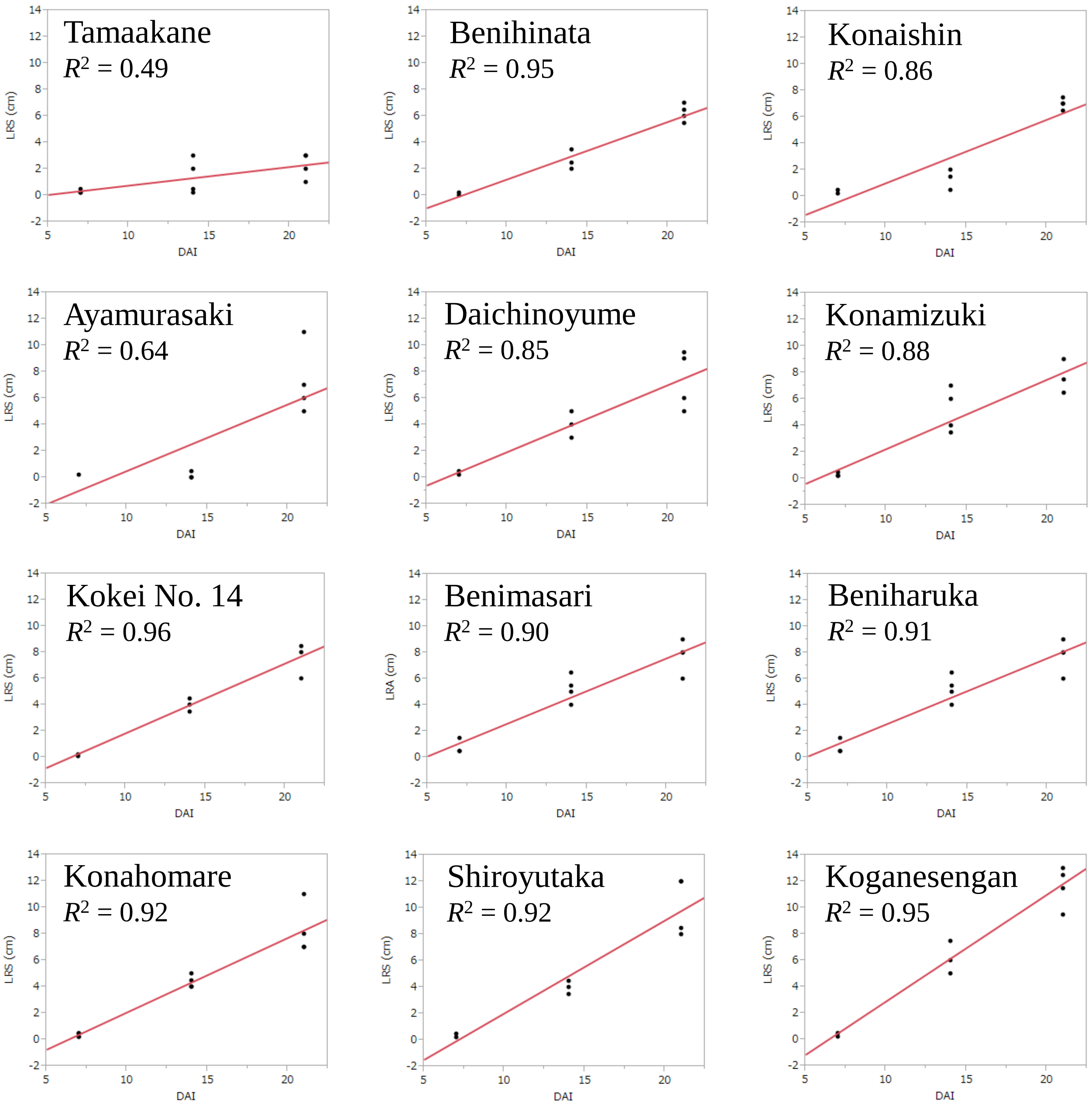

Supplemental Fig. 3 The relationship between DAI and LRS. Four cuttings of a cultivar on each of 7, 14 and 21 DAI with 10^6 conidia/ml conidial suspension. 'Benihinata' is described as 'Kyushu No. 201' at the Kyushu Okinawa Agricultural Research Center, NARO (2023).

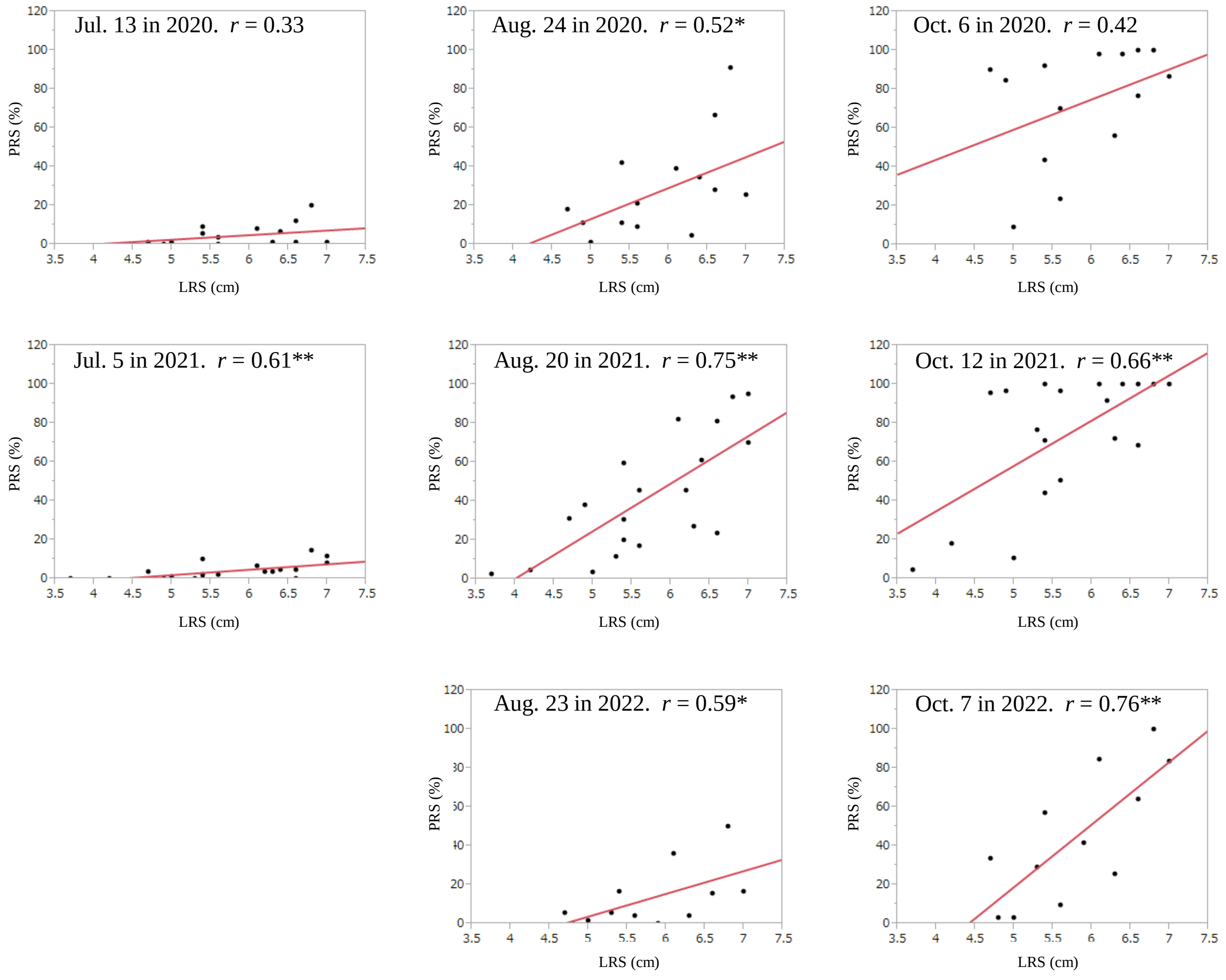

Supplemental Fig. 4 Graphs indicating the correlation between the proportion of the plants rotted at the basal part of a stem (PRS) in field tests and the length of the rotted part of a stem (LRS) in laboratory tests. *$p < 0.05$. **$p < 0.01$.
